# Supplementary material for: Disrupted cholesterol biosynthesis and hair follicle stem cell impairment in the onset of alopecia
Source: PLoS One. 2025 Sep 15;20(9):e0308455. doi: 10.1371/journal.pone.0308455 (PMC12435693; doi:10.1371/journal.pone.0308455)
Supplement: S1 File — (DOCX) [file pone.0308455.s001.docx]

Patient qRTPCR data

| **SOX9** | TF U | TF A | | DF U | DF A |  | DC U | DC A | | FD U | FD A |  | FFA U | FFA A | | LPP U | LPP A |  | CCCA U | CCCA A |
| --- | --- | --- | --- | --- | --- | --- | --- | --- | --- | --- | --- | --- | --- | --- | --- | --- | --- | --- | --- | --- |
|  | 1 | 1.6 |  | 1 | 0.4 |  | 1 | 0.23 |  | 1 | 0.6 |  | 1 | 0.612 |  | 1 | 0.79 |  | 1 | 0.26 |
|  | 0.1 | 0.1 |  | 0 | 0.07 |  | 0.04 | 0.06 |  | 0 | 0.15 |  | 0.051 | 0.159 |  | 0.06 | 0.04 |  | 0.03 | 0.05 |
|  |  |  |  |  |  |  |  |  |  |  |  |  |  |  |  |  |  |  |  |  |
|  |  |  |  |  |  |  |  |  |  |  |  |  |  |  |  |  |  |  |  |  |
| **LGR5** | TF U | TF A | | DF U | DF A |  | DC U | DC A | | FD U | FD A |  | FFA U | FFA A | | LPP U | LPP A |  | CCCA U | CCCA A |
|  | 1 | 0.7 |  | 1 | 0.29 |  | 1 | 0.28 |  | 1 | 0.29 |  | 1 | 0.67 |  | 1 | 0.83 |  | 1 | 0.44 |
|  | 0.02 | 0.1 |  | 0 | 0.09 |  | 0.06 | 0.06 |  | 0 | 0.03 |  | 0.01 | 0.02 |  | 0.03 | 0.08 |  | 0.04 | 0.02 |
|  |  |  |  |  |  |  |  |  |  |  |  |  |  |  |  |  |  |  |  |  |
|  |  |  |  |  |  |  |  |  |  |  |  |  |  |  |  |  |  |  |  |  |
|  |  |  |  |  |  |  |  |  |  |  |  |  |  |  |  |  |  |  |  |  |
|  |  |  |  |  |  |  |  |  |  |  |  |  |  |  |  |  |  |  |  |  |
|  | TF U | TF A | | DF U | DF A |  | DC U | DC A | | FD U | FD A |  | FFA U | FFA A | | LPP U | LPP A |  | CCCA U | CCCA A |
| **SHH** | 1 | 2.4 |  | 1 | 0.7 |  | 1 | 0.25 |  | 1 | 0.71 |  | 1 | 0.419 |  | 1 | 0.64 |  | 1 | 0.48 |
|  | 0.05 | 0 |  | 0 | 0.04 |  | 0.07 | 0.21 |  | 0.1 | 0.13 |  | 0.04 | 0.16 |  | 0.02 | 0.03 |  | 0.0329 | 0.0302 |
|  |  |  |  |  |  |  |  |  |  |  |  |  |  |  |  |  |  |  |  |  |
|  |  |  |  |  |  |  |  |  |  |  |  |  |  |  |  |  |  |  |  |  |
|  |  |  |  |  |  |  |  |  |  |  |  |  |  |  |  |  |  |  |  |  |
| **Wnt5A** | TF U | TF A | | DF U | DF A |  | DC U | DC A | | FD U | FD A |  | FFA U | FFA A | | LPP U | LPP A |  | CCCA U | CCCA A |
|  | 1 | 0.2 |  | 1 | 3.71 |  | 1 | 0.48 |  | 1 | 1.97 |  | 1 | 0.63 |  | 1 | 0.68 |  | 1 | 0.523 |
|  | 0.02 | 0.1 |  | 0.1 | 0.21 |  | 0.06 | 0.07 |  | 0.1 | 0.15 |  | 0.015 | 0.013 |  | 0.09 | 0.01 |  | 0.1 | 0.018 |

**In vitro Data**

LGR5

| **Control** | **DMSO** | **Ethanol** | **DHCR7** | **BM15766** |
| --- | --- | --- | --- | --- |
| 1 | 0.99 | 1 | 0.4 | 0.64 |
| 0.04 | 0.04 | 0.03 | 0.02 | 0.02 |

SOX9

| **Control** | **DMSO** | **Ethanol** | **DHCR7** | **BM15766** |
| --- | --- | --- | --- | --- |
| 1 | 1.04 | 1.04 | 0.17 | 0.57 |
| 0.05 | 0.03 | 0.04 | 0.05 | 0.01 |

SHH

| **Control** | **DMSO** | **Ethanol** | **DHCR7** | **BM15766** |
| --- | --- | --- | --- | --- |
| 1 | 1.01 | 0.97 | 0.99 | 0.62 |
| 0.02 | 0.02 | 0.04 | 0.02 | 0.04 |

Wnt5A

| **Control** | **DMSO** | **Ethanol** | **DHCR7** | **BM15766** |
| --- | --- | --- | --- | --- |
| 1 | 1.010874 | 1.07425 | 0.79795 | 1.017969 |
| 0.053871 | 0.037218 | 0.022502 | 0.034861 | 0.07599 |

In vivo data

| \| LGR5 \|  \| \| --- \| --- \| \| DMSO \| BM15766 \| \| 1 \| 0.31 \| \| 0.04 \| 0.01 \| | | |  |  |  |  |  |  |  |  |  |  |
| --- | --- | --- | --- | --- | --- | --- | --- | --- | --- | --- | --- | --- | --- | --- | --- | --- | --- | --- | --- | --- |
|  | | |  |  |  |  |  |  |  |  |  |  |
| \| Wnt5A \|  \| \| --- \| --- \| \| DMSO \| BM15766 \| \| 1 \| 0.3 \| \| 0.04 \| 0.05 \|  \| SOX9 \|  \| \| --- \| --- \| \| DMSO \| BM15766 \| \| 1 \| 0.29 \| \| 0.07 \| 0.05 \|  \| SHH \|  \| \| --- \| --- \| \| DMSO \| BM15766 \| \| 1 \| 0.47 \| \| 0.06 \| 0.02 \| | | |  |  |  |  |  |  |  |  |  |  |
|  | | |  |  |  |  |  |  |  |  |  |  |
| LGR5 |  |  |  |  |  |  |  |  |  |  |  |  |
| Ethanol | 7DHC |  |  |  |  |  |  |  |  |  |  |  |
| 1 | 0.3 |  |  |  |  |  |  |  |  |  |  |  |
| 0.04 | 0.01 |  |  |  |  |  |  |  |  |  |  |  |

| Wnt5A |  |
| --- | --- |
| Ethanol | 7DHC |
| 1 | 1.04 |
| 0.07 | 0.04 |

| SOX9 |  |
| --- | --- |
| Ethanol | 7DHC |
| 1 | 0.31 |
| 0.02 | 0.04 |

| SHH |  |
| --- | --- |
| Ethanol | 7DHC |
| 1 | 0.28 |
| 0.02 | 0.04 |

**Ex vivo data**

|  | Ctrl | VC | 7DHC | BM15766 |
| --- | --- | --- | --- | --- |
| LGR5 | 1 | 0.97 | 0.13 | 0.26 |
|  | 0.05 | 0.04 | 0.02 | 0.04 |
|  |  |  |  |  |
|  | Ctrl | VC | 7DHC | BM15766 |
| SOX9 | 1 | 0.96 | 0.21 | 0.37 |
|  | 0.02 | 0.04 | 0.03 | 0.07 |
